# Development of New Health Risk Assessment of Nanoparticles: EPA Health Risk Assessment Revised

**DOI:** 10.3390/nano13010020

**Published:** 2022-12-21

**Authors:** Michal Macko, Jan Antoš, František Božek, Jiří Konečný, Jiří Huzlík, Jitka Hegrová, Ivo Kuřitka

**Affiliations:** 1 Centre of Polymer Systems, Tomas Bata University in Zlin, třída Tomáše Bati 5678, 760 01 Zlín, Czech Republic; 2 Faculty of Logistics and Crisis Management, Tomas Bata University in Zlin, Studentské nám. 1532, 686 01 Uherské Hradiště, Czech Republic; 3 Transport Research Centre, Division of Sustainable Transport and Transport Structures Diagnostics, Líšeňská 33a, 619 00 Brno, Czech Republic

**Keywords:** health risk assessment, nanoparticles, toxicity

## Abstract

The concentration of nanoparticles in the ambient air can lead to induced toxicities; however, it appears that nanoparticles’ unique properties are completely omitted when assessing health risks. This paper aims to enhance the EPA health risk assessment by incorporating two new variables that consider the size of nanoparticles: the toxicity multiplier and the size multiplier. The former considers the qualitative aspect of the size of particles within a concentration, whilst the latter takes into account the effects associated with the number of particles of the specific *i-th* size distribution interval. To observe the impact of the new variables, a case study was performed. The studied element was cadmium, which was measured using ICP-MS to discover concentrations of size fractions, ranging from <15.1 to <9830 nm. Next, the cadmium concentration is assessed using both the current state-of-the-art method and the proposed method with adjustments. Based on the new approach, the final risk was 1.1 × 10^−5^, which was almost 24 times higher compared with the current method. The contribution of nanoparticles to the risk value grew from barely 6% to an alarming 88%. Therefore, the enhanced method can lead to more realistic results when assessing the health risks of nanoparticles.

## 1. Introduction

Air pollution is an essential factor in the quality of the environment; it has a high impact on the life of organisms and directly affects human health. It has been statistically proven that there is a relationship between ambient air pollution, cardiovascular morbidity, and mortality [1,2,3]. For this reason, many studies in the field of health risk assessment have addressed this issue, specifically on particulate matter produced by transport [4,5,6,7,8,9]; the research in this area is still receiving significant attention [10,11,12]. Nanoparticle health risks from ambient air found its attention slightly later but is also a substantial research field [13,14,15,16,17].

Nanoparticles are produced in the environment by natural or anthropogenic processes. It has been displayed that the primary source of anthropogenic atmospheric nanoparticles is transportation and the size of particles along the roads can reach hundreds of nanometers [18]. The primary sources of nanoparticles from traffic are exhaust gases, brakes, catalytic converters, and tire wear on the road [19]. Most diesel-derived nanoparticle sizes range between 20 and 130 nm and gasoline vehicles produce nanoparticles ranging from 20 to 60 nm [20]. Among other important sources, anthropogenic NPs can also stem from industrial, medical or domestic activities, high-temperature processes, solid waste incineration, smelting and welding processes (metal particles), or nanotechnology facilities that produce nanoscale products [21,22].

Nanoparticles, compared with larger 10 μm particles trapped in the upper respiratory tract or 2.5 μm particles that settle in the lungs, can penetrate the secondary organs and, therefore, may present a higher risk for human health [23,24]. Other studies supporting these phenomena have also shown that macrophage clearance mechanisms in the lung remove harmful inhaled NPs less efficiently than larger particles [17,25,26]. However, according to the literature, it is not only the size that can play a role when assessing nanoparticle toxicity. Other factors include chemical composition, shape, structure, magnetic, mechanical or optical properties, surface forms, and surface charge or agglomeration potential [27,28,29,30]. Regarding the toxicity itself, in this paper it will be understood in terms of the broadest possible definition as an ability of a substance that harms a living organism [31]. Wherever necessary, in order to compare relative toxicity, narrower definitions will be consistently considered in particular cases. 

### 1.1. Nanoparticles: Specific Toxicity and Size

The scope of this paper focuses on size as a significant parameter, as it is the size that imparts the specific nanotoxicity effects and largely determines the unique interaction of nanoparticles with living systems. One possible outcome is that nanoparticles, due to their size, can easily diffuse into the gastrointestinal tract, which can lead to the aggregation of nanoparticles in the human body and other organisms [32]. The size of nanoparticles, which range from 1 to 100 nm, can be compared with the size of protein globules (2–10 nm), the diameter of DNA helix (2 nm), or the thickness of cell membranes (10 nm), therefore allowing them to enter cells or cell organelles. A study that researched the penetration ability of different-sized particles discovered that gold nanoparticles no larger than 6 nm could enter the cell nucleus but, on the other hand, particles with a size of 10 or 16 nm could penetrate only the cell membrane and were found only in the cytoplasm. Based on this study, it can be stated that several nanometers in size can allow a particle deeper penetration, thus allowing it to cause more damage to the cell [33]. 

Another study observed the dependence of the toxicity of gold nanoparticles with sizes from 0.8 to 15 nm and the results showed a 60-times less-toxic effect of particles measuring 15 nm compared with 1.4 nm particles. The increased toxic effect was caused specifically to fibroblasts, epithelial cells, macrophages, and melanoma cells. Another important finding was that 1.4 nm nanoparticles caused cell necrosis within 12 h after they were added to the cell-culture medium, in comparison with 1.2 nm nanoparticles that caused mainly apoptosis [34]. Based on these results, it can be concluded that nanoparticles can enter the nucleus and that the size, which is similar to the size of a major groove of DNA, enables such particles to efficiently interact with the negatively charged sugar-phosphate DNA backbone, thus blocking the transcription [35,36].

### 1.2. Adverse Effects of Nanoparticles

The human body is in constant contact with the environment and, therefore, with particles from the environment. Human skin is an effective barrier to these external substances, but other uptake routes, such as oral ingestion or inhalation, are more susceptible [37]. After some particles enter the human body, they can affect multiple areas with which they come into contact, depending on their composition or size. One of the most commonly discussed irreversible type of cellular damage is oxidative stress or even organelle damage [38]. Oxidative stress results from the formation of free radicals (reactive oxygen species (ROS) and reactive nitrogen species (RNS)) caused by the activation of oxidative enzyme pathways [39]. Under prolonged oxidative stress conditions, the relative imbalance or failure of the intracellular free radical scavenger defense capability results in damage to proteins, DNA, and lipid components, dysfunction of mitochondria and endoplasmic reticulum, and ultimately apoptosis or ferroptosis [40,41,42].

However, the toxicity of some nanomaterials cannot be explained by oxidative stress due to the production of ROS [43]. Other mechanisms involved in the toxicity of nanoparticles include the release of metal ions, penetration into the cell envelope, and disruption of the cell membrane [44,45]. In some cases, the toxicity associated with the release of metal ions (especially in the case of CuO NPs) may be time-dependent, with the initial response being due to the NPs themselves and the response after 24 h being determined by the dissolved metal ions [46]. In summary, the release of metal ions is one of the most commonly recognized causes of toxicity of metal NPs that can induce genotoxic responses [47,48].

According to the thorough study conducted by Buzea et al., in 2007, and the references therein, diseases such as lung cancer, emphysema, Parkinson’s, and Alzheimer’s are associated with inhaled nanoparticles, as are conditions including asthma or bronchitis. Colon cancer and Crohn’s disease have both been connected to nanoparticles in the gastrointestinal tract. Nanoparticles that enter the bloodstream are linked to the development of heart disorders such as arteriosclerosis, blood clots, arrhythmia, and eventually cardiac death. Diseases of these organs may also result from translocation to other organs such as the liver or spleen, etc. Autoimmune illnesses such as systemic lupus erythematosus, scleroderma, and rheumatoid arthritis are also linked to exposure to certain nanoparticles. [38].

### 1.3. Quantitative Assessment of the Health Cancer Risks of Nanoparticles

As can be seen in the chapters above, there are a lot of publications that deal with nanotoxicity. On the other hand, the health risk assessment publications published nowadays mainly follow EPA guidelines in their current form, whilst nanoparticles do not receive any special attention [49,50,51,52]. Nevertheless, nanoparticle toxicity can lead to different diseases or can induce negative effects within the human body, where cancer represents one of the leading diseases that causes death. Currently, the state-of-the-art method for such calculations is based on the EPA (U.S. Environmental Protection Agency) health risk assessment, which is still being used even in recent publications [53]; for substances of categories A, B1, and B2 it is calculated as follows:

(1) ELCR=EC×IUR

where *ELCR* represents the excess lifetime cancer risk, the *EC* value (µg·m^−3^) is the exposure concentration of the substance and *IUR* represents the inhalation unit risk ((μg·m^−3^)^−1^). It is evident that this methodology treats the toxic agent in terms of the homogeneous concentration of the toxic agent (as a simple soluble compound or gas). The method does not reflect either the individual particle-related toxicity effects of the nanoparticulate matter or their quantitative toxicity related to the size of particles.

The EPA method in its current version completely omits the unique behavior of nanoparticles; even size is not considered a parameter (as will be demonstrated further in the article). This paper proposes a completely new approach to risk assessment that takes into account the size distribution of nanoparticles and the classification of their effects to provide more accurate and reliable risk results. The study provides not only a taught methodology development but also uses real data from a traffic-generated cadmium nanoparticle case study to demonstrate the importance of the novel methodology. The final draft can be used as a template or a guide for public authorities in the further development of methods intended to assess the environmental safety of different areas and to improve the identification of higher-risk zones. Subsequently, it can enhance decision-making processes when implementing risk mitigation and therefore increase overall safety.

## 2. Methods

The research had five stages. First, the samples were collected to determine the particle size distribution in the selected area in order to obtain a data set for the case study demonstrating the impact of the new methodology. The selected element for the case study was cadmium, as it falls within group B1 (probable human carcinogen) [54]. Second, the size–toxicity scale was designed to consider another variable in the calculation: the size (*D*). Third, the size-dependent classification of the adverse effects of nanoparticles according to their dependence on the particle size and the scaling rules governing the dependence of these effects on the nanoparticle size were established. As the fourth step, the size scale-dependent factors using the size distribution of the nanoparticles were implemented to the current form of EPA methodology. Finally, the health risks were calculated for the case study and the comparison between the current health risk assessment and the proposed adjusted health risk assessment was performed.

### 2.1. Case Study Sample Collection and Analysis

The sample collection for the distribution of particle size estimation was carried out in the area of the local road at the Kotlářská intersection in Brno, the Czech Republic (locality coordinates are N 49.20550°, E 16.59723°, and 230 m a. s. l.); the measurement took place from 14th November 2016 to 28th November 2016. Sampling of the ambient air, measurements of particle size distribution in real time, particle concentration in individual size fractions, and separation of size-selected particle fractions for further analysis were performed using a low-pressure impactor (ELPI + ™, Dekati Ltd., Kangasala, Finland), which allows for the selection of particles up to 14 size fractions in the 15.1–9 830 nm range.

For elemental analysis, all 14 size fractions of particles were collected using a special Whatman^®^ polycarbonate membrane. To determine the content of cadmium in the collected particles, samples were decomposed at high temperatures and pressured in ultrapure acids (HNO_3_) in closed Teflon vessels using a microwave digestion system SW-4 (Berghof, Germany). Subsequently, the digested samples were analyzed using an 8800 ICP-MS Triple Quadruple (Agilent Technologies, Tokyo, Japan). Settings of the used ICP-MS/MS were as follows: the forwarded RF power: 1550 W; carrier gas flow rate: 1.07 L·min^−1^; analyzer pressure: 2.17 × 10^−3^ Pa; integration time per isotope: 0.3 s; 3rd cell gas flow rate (ammonium in reaction MS/MS mode) + helium: 4 mL·min^−1^ NH_3_ + 1 mL·min^−1^ He. Cadmium was monitored on mass *m*/*z* 111→111. Calibration standards were prepared from a 1.0000 g·L^−1^ stock solution of single element Cd (Analytika Praha, Prague, Czech Rep.) in a concentration range of 0–10 µg·L^−1^ in a matrix of 2% HNO_3_. The internal standard solution was prepared from a 1.0000 g·L^−1^ stock solution of single elements In and Tb (Analytika Praha, Prague, Czech Republic) by mixing, in a concentration of 10 µg·L^−1^ in a matrix of 2% HNO_3_. The tuning solution 1 µg·L^−1^ was prepared from a 10 mg·L^−1^ stock solution (Agilent Technologies, Santa Clara, CA, USA (elements Ce, Co, Li, Mg, Tl, Y)) in a matrix of 2% HNO_3_. It was tuned for suitable sensitivity and robustness, intensity for Co(NH_3_)_2_ *m/z 93* and ^205^Tl, RSD% for masses, and oxide ratio (^156^CeO^+^/^140^Ce^+^), and doubly charged ions ratio (^140^Ce^+^/^70^Ce^++^) were checked. De-ionized ultrapure water (Merc Millipore, Bourlington, MA, USA) and sub-boiled nitric acid were used for the preparation of all solutions.

The measurements of the cadmium concentration were carried out according to the stringent standard EN 14902: 2005 [55].

Quality assurance (QA) and quality control (QC) were carried out using the ERM–CZ120 Fine Dust (PM10-like, European Commission’s Joint Research Centre), where the certified value for cadmium is found. ERM was decomposed, together with the samples. SRM 1640a Trace Elements in Natural Water (National Institute of Standards and Technology) were used for verifying the calibration and setting of the instrument.

From the measured concentrations, cadmium was selected for the demonstration of the new approach as it falls in the category of probable human carcinogen, which makes it suitable for EPA carcinogenic risk assessment.

Since only one set of data was obtained from the single sample collected at the locality, there is no average or standard deviation based on between sample comparison available. On the other hand, the continuous collection of the sample took fourteen days, representing thus a two-week average in atmospheric particle concentration, whereas the uncertainty of the single cadmium concentration measurement was estimated up to 20% using repeated analysis of standards by the method described above. Therefore, the values of the obtained results were rounded to two digits.

### 2.2. Quantitative Assessment of the Health Cancer Risks

The state-of-the-art assessment of health risks is performed by the valid methodology of the Ministry of the Environment of the Czech Republic for carcinogenic risk assessment [56], which is fully compliant with the EPA assessment. The data processing and interpretation is identical to the EPA assessment method as described in the first two paragraphs of the Section 1.3.

The development of the new method for the NP health risk assessment is the main result of this work and is described in the following Section 3.1.

## 3. Results

### 3.1. Development of a New Method for the NP Health Risk Assessment

This chapter aims to describe the approach taken when designing the new parameters that are currently not considered when performing the health risk assessment. Specifically, attention will be put into the current approach of modeling the toxicity of nanoparticles, followed by the design of two new variables—toxicity multiplier (*TM*) and size multiplier (*SM*)—that will be incorporated into the final calculation.

#### 3.1.1. Draft of Size–Toxicity Scale Relationship

**Figure 1:**
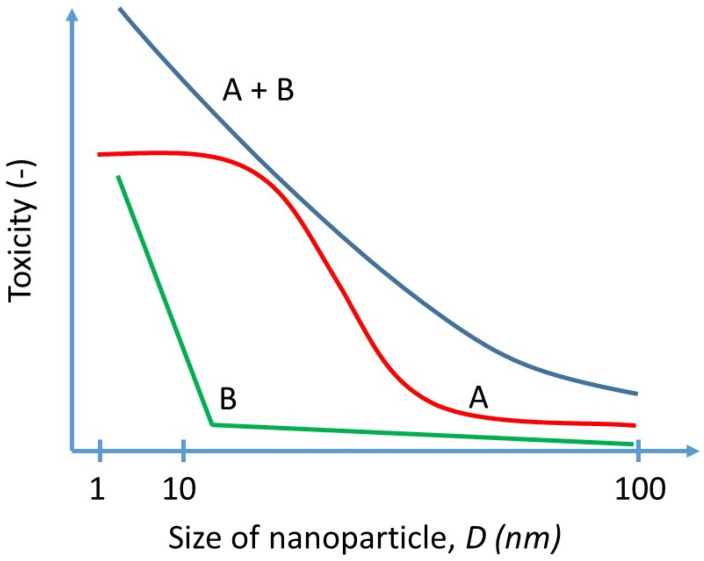
Theoretical estimate of the size and toxicity relationship. A: sigmoidal curve; progressive dependence of toxicity with decreasing size down to a certain saturation limit. B: threshold curve; a small change of the particle size results in a large increase of toxicity below a certain critical size (a threshold). A + B: synthesis of both approaches; the toxicity grows with decreasing particle size. Note: the toxicity axis has no unit in this graph as it is a tentative scale for displaying general trends in toxicity change with NP diameter.

Currently, a theory behind the size–toxicity relation of nanoparticles can be modeled in two ways, as shown in Figure 1 [27]. Approach A suggests a sigmoidal relationship that changes with saturation. On the other hand, Approach B suggests a specific size threshold that significantly increases toxicity. The idea of Approach A suggests that after some saturation, the toxicity cannot be increased anymore but, based on various research as demonstrated further, it was observed that smaller particles could induce larger toxicities. The argument for Approach B could be that some particle systems may have more than one threshold, e.g., larger particles are prevented from entering the organism at the respiratory tract, particles ~50 nm are blocked from entering the body via skin pores although some nanoemulsions with particle size of 80 nm can diffuse into but not penetrate the viable epidermis [57], whereas smaller particles ~10 nm can translocate via blood membranes, which will result in different thresholds and potential toxicities. In our opinion, both approaches relate to specific effects of nanoparticle toxicity; yet, a better explanation could be found in synthesizing these two approaches, as the particles of the same material composition may have more than one effect mechanism depending on the size in either of the two ways. As a result, a simple slope line is obtained. The scale ranges from 1 nm to 100 nm, as this meets the definition of nanoparticles according to both the EU and FDA [58,59].

The proposed scale considers both models and, rather than being based on concrete evidence and specific relationships, a simplified general approach is adopted. Many studies observe and point out a dependence on size and toxicity, but the research performed contains a lot of “noise”, i.e., different elements, surface charges, culture mediums, or test media. Therefore, the only observed parameters for drafting such a scale were size and toxicity, which allow for a reduction of all the overwhelming details blurring the general trend. The analysis of current results from various articles focused on size–toxicity dependence was performed and the outcome is displayed in the following Table 1.

Table 1 contains a summary of studies in various articles that deal with size-dependent toxicities and, whilst it is not possible to determine the exact multiplier between sizes (as it appears to be heavily determined by other factors such as the substance, test subject, culture, zeta potential, and others), it is possible to perform simple comparisons between specific sizes and their toxicities in the respective publications. Based on that, the trend that smaller particles induced higher toxicities is observed.

**Figure 2:**
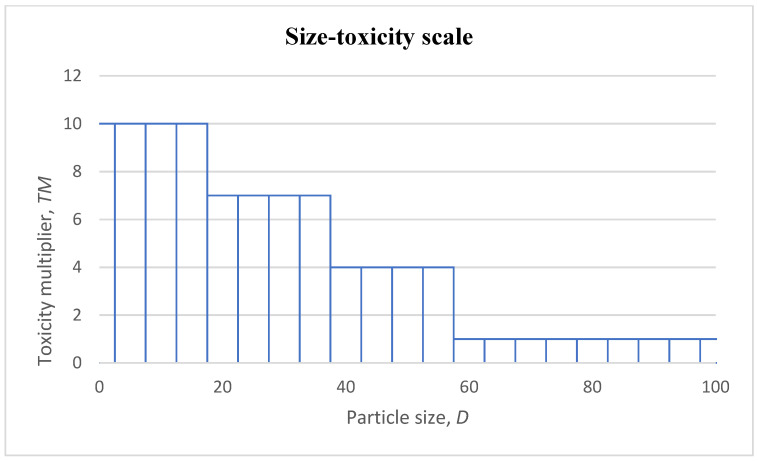
Size–toxicity scale design.

As can be seen, the toxicity of particles decreases with an increased particle size in all selected cases. It is still difficult to determine the exact thresholds for the size–toxicity relationship, but a simplified scale can be deduced using the theoretical estimate shown in Figure 1 and the results from Table 1. The relative increase of toxicity of an individual particle is expressed as a toxicity multiplier (*TM*), with dependence on the particle size in Figure 2. The scale has the following multiplicators: 1, 4, 7, and 10. The particles above 60–100 nm are multiplied by one, based on the study, where it was displayed that between 80 and 100 the resulting toxicity was similar between these groups and, at the same time, not significant compared with the smaller sizes [69]. Due to this study, along with the lack of other data, the particles of sizes from 60 to 100 nm are considered as not having increased toxicity levels, as it may be expected that they have no specific difference in nano size-related effect. On the other hand, the multiplicator of 10 is used for particles lower than 20 nm, as the highest toxicities are found below the 20 nm threshold.

It is not excluded that particles on a micrometric (>100 nm) scale might have an increased risk as well. Nevertheless, the bigger particles without any nano size-related effect shall be most reasonably treated by a *TM* value of one as their size does not invoke any specific nanotoxicity effect. In other words, let the simplest assumption be that they comply with the unspecific value of IUR.

#### 3.1.2. Draft of Size–Distribution Toxicity Relationship

There are still uncertainties associated with many factors related to the properties of nanoparticles, organisms’ responses to nanoparticles, and, to date, unknown toxic properties of nanoparticles due to a small number of studies performed or due to insufficient technology [72]. Nevertheless, according to contemporary understanding, the NP properties that must be considered when assessing hazards are the following: size, shape, composition, and surface characteristics [73]. In fact, the composition, shape, and surface characteristics are assumed as given parameters just by choice of the nanoparticle type, i.e., by its definition, when assessing the toxicity of a specific type of nanoparticle. Hence, the main characteristic that plays a role in the nanoparticle effect is size. In nanoscience, the investigation usually starts from the bulk and proceeds to smaller things. We discussed emerging effects on the specific nano size thresholds in the previous section and developed a toxicity multiplier (*TM*) scale based on these non-scaling parameters. This seems like a contradiction, but, nonetheless, it is a qualitative assessment scale. Unlike *TM* above, here we develop a size multiplier (*SM*), which is based on the size-dependent effects of parameters with inherent scalability that follow corresponding scaling laws as the particles become smaller.

Therefore, the NP-induced toxic mechanisms must be reclassified concerning the parameters following the scale laws. The reclassification is based on a comprehensive summary of the most common mechanisms of effects elicited by NPs in a study by Sukhanova et al. and references therein [74]. Some mechanisms may belong to more than one group, as various characteristics of nanoparticles may become important at each step of the mechanisms:

1 Point effects occur when a single particle causes a localized effect or a series of effects or triggers a cascade of effects. All direct effects, such as the following:

- NPs may damage cell membranes by perforating them;
- NPs damage components of the cytoskeleton, disturbing intracellular transport and cell division;
- NPs disturb transcription and damage DNA, thus accelerating mutagenesis;
- NPs damage mitochondria and disturb their metabolism, which leads to cell energy imbalance;
- NPs interfere with the formation of lysosomes, thereby hampering autophagy and degradation of macromolecules and triggering apoptosis;
- NPs cause structural changes in membrane proteins and disturb the transport of substances into and out of cells, including intercellular transport;
- NPs that activate the synthesis of inflammatory mediators by disturbing the normal mechanisms of cell metabolism, as well as tissue and organ metabolism, belong to this group.
- These effects scale with the total number of particles in the organism.

2 Surface effects. The effect depends on the surface area of the particle either due to a specific surface catalytic mechanism of toxic agent production or another specific interaction between the nanoparticle surface and the biological surfaces, e.g., adherence of nanoparticles to the membranes.

- NPs may cause oxidation via the formation of ROS and other free radicals;
- NPs may damage cell membranes by perforating them;
- NPs interfere with the formation of lysosomes, thereby hampering autophagy and degradation of macromolecules and triggering apoptosis;
- Short-term NP effects related to the leakage of free ions of metals contained in their cores, such as cadmium, lead, and arsenic, upon oxidation by environmental agents. The larger the surface of the NPs, the faster the ion release is.
- These effects scale with the total surface of NPs present in the organism.

3 Volume effects.

- Long-term NP effects related to the leakage of free ions of metals or other toxic agents contained in their cores, such as cadmium, lead, and arsenic, upon oxidation by environmental agents or simple dissolution. If the dissolution time is long enough, the final dose of the ions depends on the total volume of nanoparticles only. These effects also include oxidative stress caused by some ions.
- This effect scales with the total volume of NPs present in the organism.

The classification is based on the total number, surface, and volume of the NPs. Let us assume the density of NPs is constant, i.e., a non-scaling parameter remaining constant for each NP concentration expressed in mass unit per volume, as in *EC*. Then, the total volume of the nanoparticles (TVNP) scales linearly with mass concentration *EC* and the size of particles plays no specific role, i.e., TVNP ∝ *EC*. The total surface area of the nanoparticles (TSANP) scales with the reciprocal second power of the particle size TSANP ∝ *EC* ∝ *D*^−2^, which means that 10 times smaller particles have a 100 times larger total surface at the same *EUC*. The total number of NPs (TNNP) scales with the reciprocal third power of the particle size TNNP ∝ *EC* ∝ *D*^−3^, which means that there are 1000 times more particles in a volume unit if they are 10 times smaller at the same EUC. The size multiplier (*SM*) is estimated as a sum of the three contributions:

(2) $$SM={\left(\frac{100\;\mathrm{nm}}{D}\right)}^{3}+{\left(\frac{100\;\mathrm{nm}}{D}\right)}^{2}+1$$

At first look, the TNNP effect outweighs the other two effects, as the TSANP effect represents together only 10% of the TNNP effect and TVNP is negligible for particles of 10 nm in size. Therefore, the size multiplier (*SM*) for very small particles can be estimated just by a simplified formula:

(3) $$SM_{D\leq 10\;\mathrm{nm}}={\left(\frac{100\;\mathrm{nm}}{D}\right)}^{3}$$

yielding round *SM_D≤_*_10 nm_ values from 1 for 100 nm NPs up to 1,000,000 for 1 nm NPs. On the other hand, it would underestimate the values for bigger particles. Therefore, the original Equation (2) is used for calculations. Moreover, the value of *D* is estimated as the center of the size fraction between its upper and lower size limit. The lowest size limit for particle capture in the used low-pressure impactor is declared at 6 nm.

#### 3.1.3. Draft of a New Quantitative Assessment of the Health Risks of Nanoparticles

In order to include the effect of nanoparticle size distribution, the overall excess lifetime cancer risk (ELCR) value for substances of categories A, B1, and B2 is calculated as:

(4) $$ELCR=\overset{h}{\underset{i=1}{\sum }}ELCR_{i}\;$$

where $ELCR_{i}$ is the excess lifetime carcinogenic risk of the *i*-th size group for the *h*-th number of size groups. To calculate the value of $ELCR_{i}$, the observed concentration of the substance in the air and the inhalation unit risk (IUR) value is multiplied:

(5) $$ELCR_{i}=EC_{i}\times IUR$$

IUR is the inhalation unit risk ((μg·m^−3^)^−1^), corresponding to the upper limit of the lifetime risk probability of developing cancer with a sustained exposure of 1 µg·m^−3^ air [75]. The $EC_{i}$ value (µg·m^−3^) is the exposure concentration of the substance for the *i*-th size group and is calculated as follows:

(6) $$EC_{i}=\frac{CA_{i}\times ET\times EF\times ED}{AT}\;$$

where $CA_{i}$ is the concentration of nanoparticles of the *i*-th size group (µg·m^−3^), ET means the exposure time for which the subject is exposed to the substance per day (h·day^−1^), EF stands for the exposure frequency on the days when the subject is exposed to the contaminant within the given year (day·year^−1^), ED is the duration of substance exposure during the life (year), and AT means the averaging time when the subject is exposed to the pollutant (h), according to the following formula:

(7) AT=ED×365×24

#### 3.1.4. Implementation of Toxicity and Size Multipliers

Having already included the effect of nanoparticle size distribution, the overall excess lifetime cancer risk is further developed:

(8) $$ELCR_{i}^{TMSM}=EC_{i}\times IUR\times TM_{i}\times SM_{i}$$

where $ELCR_{i}^{TMSM}$ is the excess lifetime cancer risk for the *i*-th group, that is calculated as $EC_{i}$, which is the exposure concentration of each *i*-th group, multiplied by the inhalation unit risk IUR ((μg·m^−3^)^−1^) for the specific substance, multiplied by $TM_{i}\;\mathrm{and}\;SM_{i}$. The toxicity multiplier is based on the corresponding qualitative assessment of the size impact on the toxicity effect, as seen in Figure 2, and the size multiplier is based on the particle size within the specific *i*-th group. Then, the total excess lifetime cancer risk for the measured concentration, also considering the size of nanoparticles, is calculated as follows:

(9) $$ELCR^{TMSM}=\overset{h}{\underset{i=1}{\sum }}ELCR_{i}^{TMSM}$$

where the sum of individual $ELCR_{i}^{TMSM}$ results in the combined toxicity $ELCR^{TMSM}$.

### 3.2. Case Study: Demonstration of Traffic Generated Cadmium Nanoparticle Health Risk Assessment

The demonstration of the new approach will be displayed on the samples collected as described in Section 2.1.

#### 3.2.1. Health Risk Assessment Procedure

To calculate the health risks, a group of people who live and work in the area of the sample collection and thus are in contact with the substance for 24 h (value ET), has been considered as input data. Using the chronic exposure calculation, the exposure duration ED was defined by the average life expectancy, which is currently estimated to be 78.33 years [76]. Following the set scenario, the EF value was set to 365 days per year. The averaging time AT was calculated using Equation (7) and the value of 686,170.8 was obtained. Next, it was necessary to determine the $EC_{i}$ value, according to Equation (6), which in this case is equal to the $CA_{i}$ value due to simplifying the given fraction. The calculation of the lifetime carcinogenic risk value $ELCR_{i}$ was performed by using Equation (5).

According to the EPA, the IUR value for cadmium is set to 1.8 × 10^−3^ (μg·m^−3^)^−1^. The US EPA acceptable risk value ranges from 10^−4^ to 10^−6^ in relation to substance concentration. Thus, it can be stated that the inequality *ELCR* ≤ 10^−4^ must be met in the case of risk acceptance [54]. The last performed step is that each particle group based on its size is multiplied using the TM and SM and the final $ELCR^{TMSM}$ value was calculated.

#### 3.2.2. Assessment of the Distribution of Cadmium Pollutant in the Air Depending on the Diameter of Solid Particles

The measured concentration of cadmium particles in individual size groups is shown in Table 2. The collected sample shows characteristics of the centrally symmetric particle distribution, with concentration values ranging from 0.45 to 69 pg·m^−3^, with a peak at the particle size of 379 nm. As can be seen, the population of cadmium nanoparticles is mostly represented by fractions ranging from 254 to 942 nm particles, representing more than 80% of the total concentration. The total cadmium concentration is 260 pg·m^−3^, where the cadmium nanoparticle concentration is only 15 pg·m^−3^, which is equal to nearly 5.9% of the measured concentration. The nominal NP size distribution is based on data obtained by the low-pressure impactor instrument, which is generally found appropriate for airborne nanoparticle analysis but is not intended for the subtle distinguishing between single particles and their aggregates or agglomerates. On the other hand, it provides good agreement between the aerodynamic diameter and the particle diameter as demonstrated, e.g., by Noël et al., in 2013 [77]. Moreover, the robustness of the proposed new method does not require such levels of detail, as it does not consider the eventual deagglomeration or disaggregation of particles in the human body.

#### 3.2.3. Quantification of Health Risks of Cadmium Particles and Evaluation of the New Methodology

Table 3 shows the partial and final results of the performed health risk assessment, where the collected data were divided into size groups, where three groups belonged in the range of nanoparticles, and particles with a size larger than 95.2 nm were summed into one group, because the toxicity multiplier is equal to one. As can be seen, the $ELCR_{i}$ values correspond to the $EC_{i}$ values, as expected, with the highest value of 4.4 × 10^−7^ for the particle size group 95.2–9830 nm and the lowest value of 8.1 × 10^−10^ for the group with the edge 15.1 nm.

Using Equation (4), the specific risks for each size group and the lifetime carcinogenic risk for the measured cadmium concentration are summed, taking into account the size-related toxicity. To demonstrate the incorrectness of the current health risk assessment and to display the impact of the new methodology, a comparison is performed, as can be seen in Table 4. In both cases, the same scenario regarding exposure time is considered, which results in the situation where EC=CA. If the current health risk assessment method were used, by applying Equation (1) the impact of nanoparticles on the total risk will also be only 5.9%, as it is the ratio of nanoparticles in the total concentration. That will indicate that nanoparticles are actually neglected, despite the fact that numerous studies show the increased toxicities that are dependent on their sizes. By applying the new methodology, there is an evident increase in the calculated risk value by approximately 24 times. However, the resulting risk values are still profoundly below the acceptance level and there is no reason to begin with risk mitigation activities, which is a good message for the population in Brno city. In any case, risk monitoring should be carried out even if the risk at the moment is at the acceptance level (because of its variability over time); especially, this can be helpful wherever the concentration of (nano)particles is higher. Last but not least, an evident result is the increase of the nanoparticles’ share on the final health risk values, which has changed from 5.9% to 88% in comparison with the current method, confirming how severe the underestimation of nanoparticle fractions by the current EPA method is.

Some recent studies, as demonstrated in the specific situation of the health risk assessment of asbestos in the air, claim that the EPA methodology overestimates the risk [78] or is unnecessarily conservative [79]. Indeed, it can be argued that the EPA methodology [80] is too general and uses unit risk factors based on similarity rather than individual factors applying to each specific situation. Starting from the same critical standpoint, we derived completely different conclusions. Direct evidence for health risk factor exposure levels and their effects is rarely ever complete, therefore assumptions need to be made in arriving at the risk estimate [81]. It must be emphasized that the purpose of risk assessment is primarily to support decision making during uncertainty and preventive action rather than mathematical prediction of a real health outcome [82]. According to our opinion, based on the presented work, the standard EPA methodology significantly underestimates the risk of nanoparticles by not considering the particle size distribution and its impact on the toxicity of NPs. Therefore, the proposed toxicity multiplier and size multiplier represent the first necessary steps in designing a comprehensive method for assessing the health risks of nanoparticles. On the other hand, we are fully aware of the limits of our work. The case study used to demonstrate our new methodology approach is based on one toxic element and one location. To better develop the EPA-based health inhalation risk assessment, future studies aimed at the collection of more experimental values and metanalyses should include various representatives to cover the plethora of airborne nanoparticulate pollutants.

## 4. Conclusions

The method developed by EPA in the past is still being perceived as state-of-the-art and is used when performing health risk assessments, even though it appears that the effects and unique attributes of nanoparticles are not considered. Based on various research, it is displayed that nanoparticles can induce more severe toxicity compared with larger particles, specifically due to their smaller size. Based on that, the aim of this article was to enhance the current approach by developing new variables that would incorporate the size of nanoparticles into the formula, as it was this factor that was defined as the significant parameter when assessing toxicity.

The basis for the development of the new variables was taken from the current model that describes two different approaches to the size–toxicity relationship, whereas Model A describes continuous toxicity increase as size decreases and Model B describes the threshold level, where the toxicity is rather stagnant, but after reaching the threshold it increases considerably. By our understanding, the more fitting model would be based on the synthesis of these two models, as there are more thresholds that could trigger increased toxicity due to the composition of the human body, e.g., pores on the skin ~50 nm or cell membranes of 10 nm.

The first variable that was suggested is the toxicity multiplier (*TM*), which tries to fit different thresholds that were set to 20, 40, and 60 nm. The basis was taken from different research where similar thresholds were used to observe the effect of size to the final toxicity. The toxicity multiplier adds another multiplication based on the measured size as follows: 1× multiplier for 60–100 nm, 4× multiplier for the range between 40 and 60 nm, 7× multiplier for the range between 20 and 40 nm, and 10× multiplier for particles between 1 and 20 nm. Thus, the *TM* is a qualitative scale for the NPs’ impact depending on their size.

The second variable that was suggested is the size multiplier (*SM*) that takes into account the particle size as an inherently scalable measure of the particle effect at a given total concentration. Besides the minor contribution of the total volume and the more important contribution of the total surface, the most important contribution to *TM* is the total number of all particles that exists within the size group of every particle, as every single particle can trigger adverse effects. The total number of particles, and therefore the *TM* as well, scales with a reciprocal third power of the particle size. By the given range from 1 to 100 nm, there will always be 1,000,000 more 1 nm particles or 1000 of 10 nm particles compared with one 100 nm particle, which must be taken into account.

To demonstrate the impact of these two new variables, we have used the previously measured cadmium concentrations that were used for the case study, and a comparison of the final results of the current method and the adjusted method was performed. Using the current method, the nanoparticles’ contribution to the final health risk assessment would be only 5.9%, as that was their volume ratio in the total concentration. By implementing new variables, we have managed to consider both the qualitative and quantitative aspects of the size of nanoparticles within the measured concentration, which has led to the significant change from the former 5.9% to 88% nanoparticle contribution, as well as an almost 24-fold increase of the final health risk assessment. Nonetheless, both final risk values are well below the acceptance level, therefore there is no need to reduce the risk in the place where the case study measurement was carried out.

## Figures and Tables

**Table 1:** Comparison of size–toxicity results from different publications.

Article	NP Size (nm)	Toxicity			Source
Pan et al., 2007	1.4	highest			[34]
	15			lowest	
Carlson et al., 2008	15	highest			
	30		lower		[60]
	55			lowest	
M.V.D.Z. Park et al., 2011	20	highest			[61]
	80		lower		
	110			lowest	
Kim et al., 2012	10	highest			[62]
	50		lower		
	100			lowest	
Passagne et al., 2012	20	highest			[63]
	100			lowest	
Y.-H. Park et al., 2013	20	highest			[64]
	100			lowest	
Huo et al., 2014	2	highest			[33]
	6	highest			
	10		lower		
	16		lower		
Seiffert et al., 2015	20	highest			[65]
	110			lowest	
Lee et al., 2016	5	highest			[66]
	100			lowest	
Cho et al., 2018	10	highest			[67]
	60		lower		
	100			lowest	
Carnovale et al., 2019	25–50	highest			[68]
	50+		lower		
Cunningham et al., 2021	20	highest			[69]
	40		lower		
	60		lower		
	80		lower		
	100			Lowest	
H. Liu et al., 2021	20	highest			[70]
	100			lowest	
Z. Zhang et al., 2022	15	highest			[71]
	50			lowest	

**Table 2:** Distribution of cadmium concentration on the aerodynamic diameter of particles (the values are not rounded but intentionally left as obtained, as only one data collection was performed).

Particle Size (nm)	Concentration (pg·m^−3^)	Particle Size (nm)	Concentration (pg·m^−3^)
15.1	0.45	599	65
29.4	1.0	942	35
53.9	4.3	1620	11
95.2	9.5	2460	4.3
154	16	3640	2.0
254	39	5340	1.1
379	69	9830	0.73

**Table 3:** Input data for health risk calculation.

Size Group (nm)	$CA_{i}\;(µg\cdot m{-}^{3})$	$EC_{i}\;(µg\cdot m{-}^{3})$	$ELCR_{i}$	TM	SM	$ELCR_{i}^{TMSM}$
15.1	4.5 × 10^−7^	4.5 × 10^−7^	8.1 × 10^−10^	10	942	7.63 × 10^−6^
29.4	1.0 × 10^−6^	1.0 × 10^−6^	1.8 × 10^−9^	7	112	1.41 × 10^−6^
53.9	4.3 × 10^−6^	4.3 × 10^−6^	7.7 × 10^−9^	4	21	6.38 × 10^−7^
95.2	9.5 × 10^−6^	9.5 × 10^−6^	1.7 × 10^−8^	1	5	8.91 × 10^−8^
95.2–9830	2.4 × 10^−4^	2.4 × 10^−4^	4.4 × 10^−7^	1	3	1.32 × 10^−6^

**Table 4:** Comparison of health risk calculation methods.

Method	Health Risk of Measured Cadmium Concentration	Nanoparticles’ Share on Health Risk Value	Nanoparticles’ Share
Standard method *ELCR*	4.7 × 10^−7^	2.7 × 10^−8^ *	5.9% *
$\mathrm{Novel}\;\mathrm{method}\;ELCR^{TMSM}$	1.1 × 10^−5^	9.8 × 10^−6^	88%

* Note: this is a hypothetical calculation for TM = 1 and SM = 1. The share of nanoparticles corresponds simply to their percentage in total cadmium concentration.

## Data Availability

Not applicable.
